# How to Detect Meniscal Ramp Lesions Using Ultrasound

**DOI:** 10.1016/j.eats.2021.02.022

**Published:** 2021-05-17

**Authors:** Junsuke Nakase, Kazuki Asai, Rikuto Yoshimizu, Mitsuhiro Kimura, Hiroyuki Tsuchiya

**Affiliations:** Department of Orthopaedic Surgery, Graduate School of Medical Sciences, Kanazawa University, Kanazawa, Japan

## Abstract

The clinical importance of meniscal ramp lesions in patients with anterior cruciate ligament tears has emerged. However, the diagnostic accuracy of magnetic resonance imaging for detecting meniscal ramp lesions is low. The advantage of ultrasonography is that it can be performed in any position and is a real-time imaging modality. The goal of this Technical Note is to describe in detail the ultrasound technique that we use to detect meniscal ramp lesions in patients with anterior cruciate ligament tears. The semimembranosus muscle is a reliable landmark for this technique. The examination position is prone, with the knee joint flexed to 70°. The most important part of this technique is to instruct the patient to perform isometric contractions in knee flexion with the support of an assistant. The presence or absence of a meniscal ramp lesion can be diagnosed preoperatively by setting the probe above the semimembranosus tendon.

The incidence of meniscal ramp lesions has been reported to be 16% to 24% of all anterior cruciate ligament (ACL) tears,^1^ and they are an important issue for knee surgeons. The term “ramp lesion” was coined by Strobel in 1988.^2^ Ramp lesions are defined as posterior longitudinal tears at the meniscocapsular junction and/or the meniscotibial ligament. Ramp lesions are sometimes referred to as ‘‘hidden lesions” because their posterior location behind the medial tibiofemoral joint often makes them hard to detect using standard anterior arthroscopic techniques. Furthermore, they are very hard to identify using preoperative conventional magnetic resonance imaging (MRI) because of its low sensitivity. In a recent review and meta-analysis, the sensitivity of MRI for ramp lesions was 0.71 (95% confidence interval 0.59-0.81), and the specificity was 0.94 (95% confidence interval 0.88-0.97).^3^ This is partly because most MRI scans are performed with the knee in near full extension, which reduces the meniscocapsular gap and can lead to false-negative results. However, MRI with the knee in a flexed position improves the sensitivity and specificity of the detection of meniscal ramp lesions compared with those of MRI with the knee in near full extension.^4^

The distal semimembranosus complex, especially the capsular branch, has been implicated in ramp lesion pathogenesis owing to its attachment to the posterior horn of the medial meniscus (PHMM). This has been demonstrated on gross^5^ and arthroscopic^6^ anatomical studies. On arthroscopic evaluation, the application of a load to the semimembranosus tendon resulted in posterior translation of the PHMM and stretching of the meniscocapsular region.

To evaluate the presence or absence of meniscal ramp lesions preoperatively, it is necessary to examine the knee in the flexed position with the semimembranosus muscle contracted, and the most suitable device is ultrasonography (US). To the best of our knowledge, there have been no reports of US observation of meniscal ramp lesions. This report is the first to demonstrate the dynamic evaluation of meniscal ramp lesions with US. The goal of this Technical Note is to describe in detail the US technique that we use to detect meniscal ramp lesions in patients with ACL tears.

## Ultrasound Technique (With Video Illustration)

**Fig 1:**
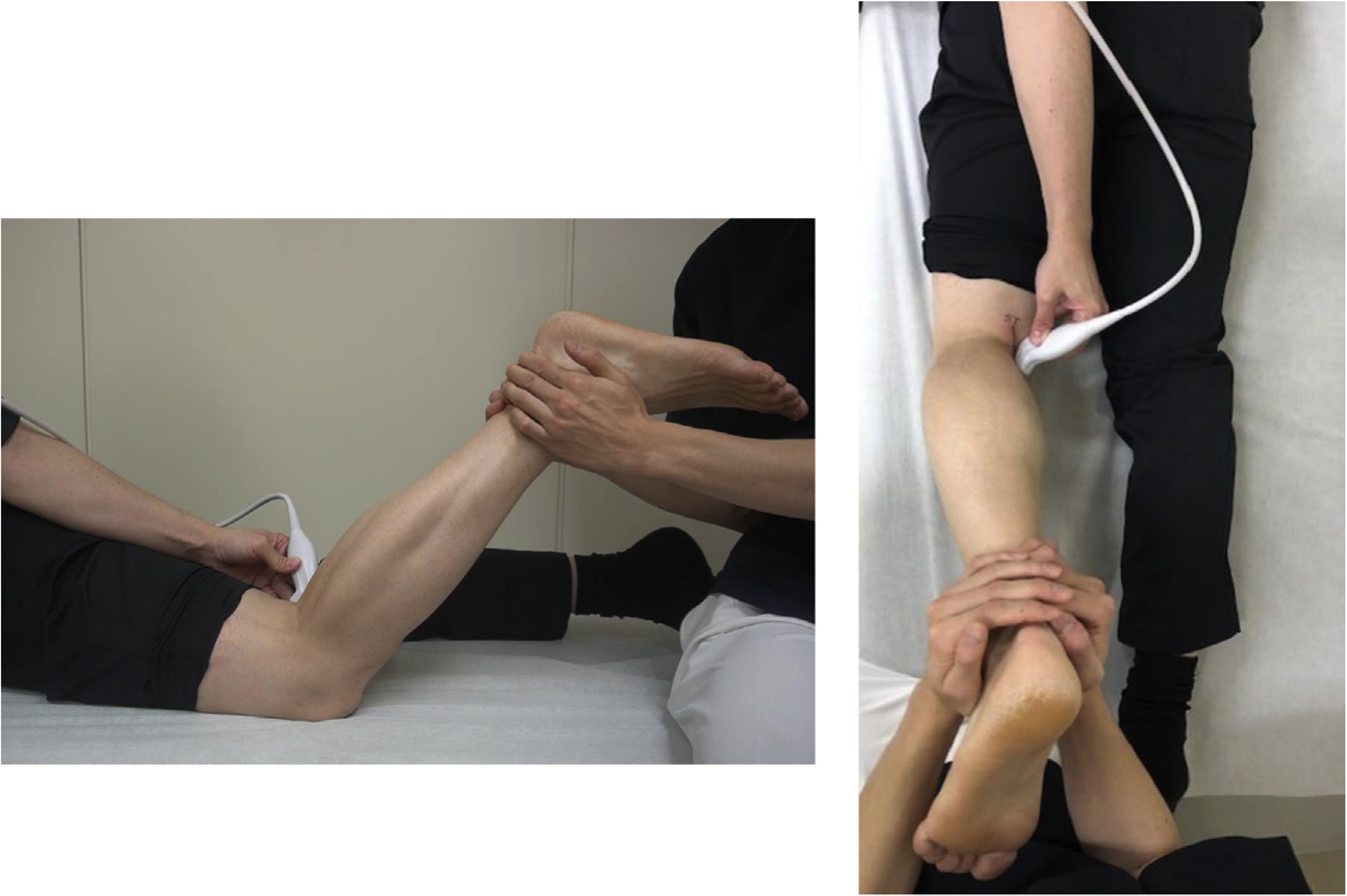
Patient position. The patient is placed in the prone position with knee flexed to approximately 70° and supported by an assistant.

**Fig 2:**
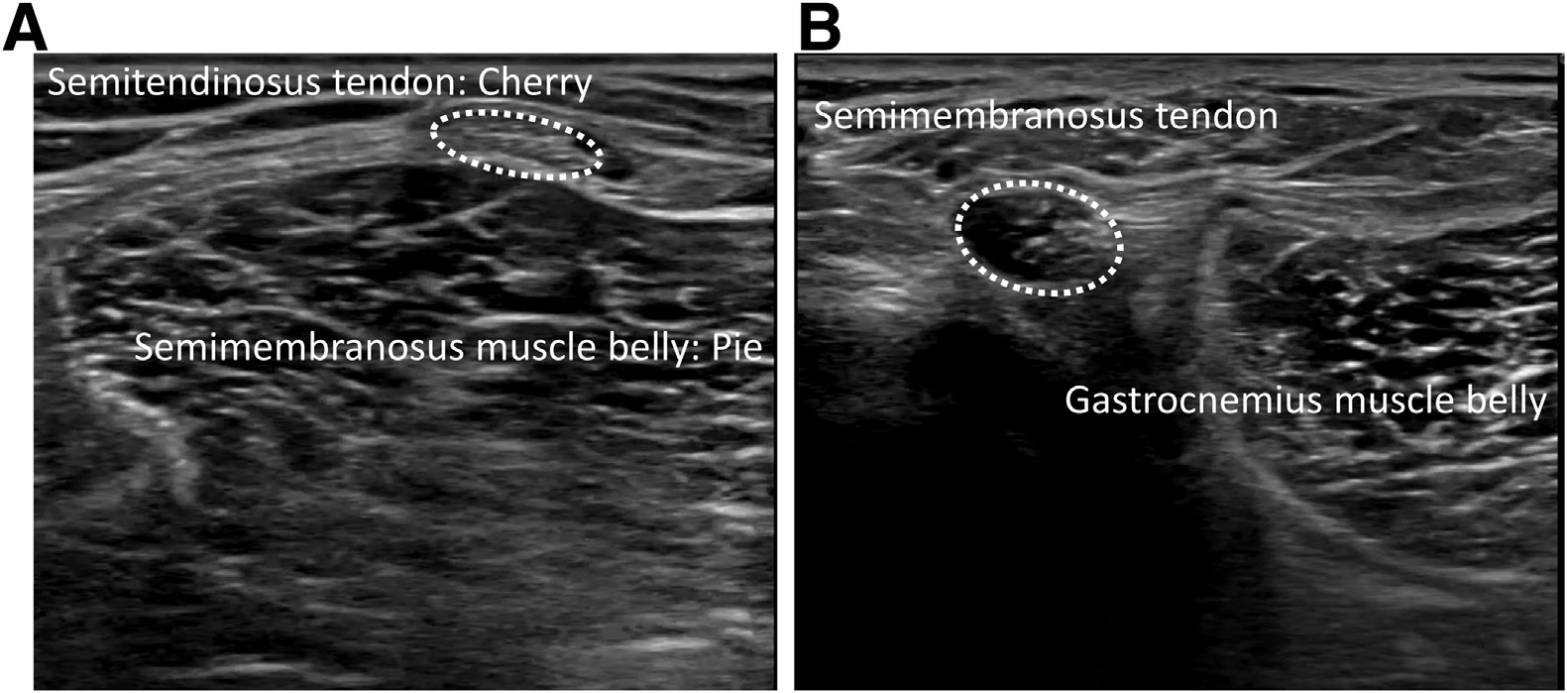
Short-axis ultrasound image at 1 cm above the knee joint line (A) and joint line (B) semitendinosus tendon overlying the semimembranosus muscle belly (left knee). This results in a “cherry on a pie” appearance (A). On the lateral side of the semimembranosus tendon, the muscle belly of the medial head of gastrocnemius is observed (B).

**Fig 3:**
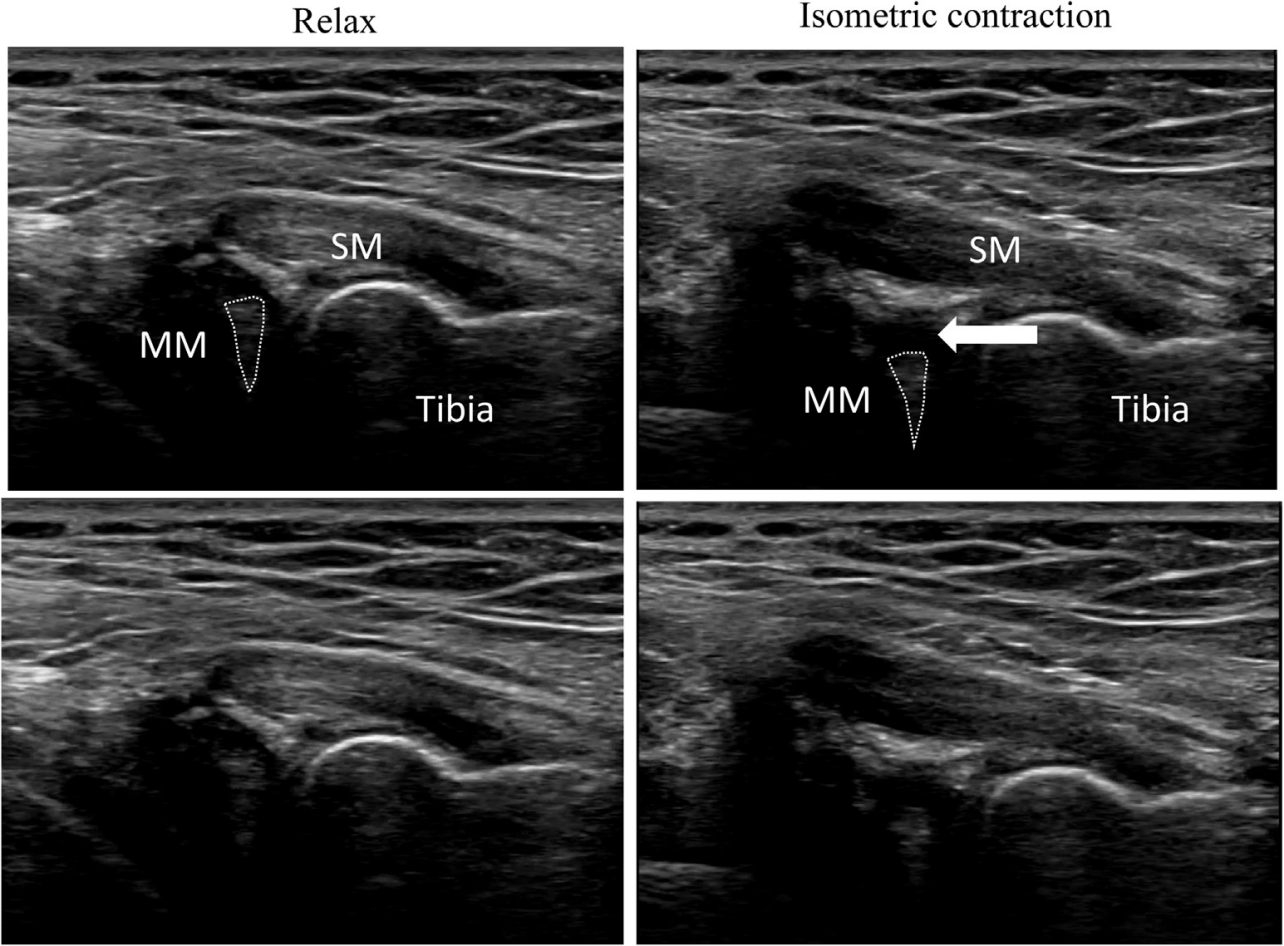
Long-axis ultrasound image at the knee joint line. The probe is placed at approximately 90° just above the semimembranosus tendon to obtain a long-axis view. Semimembranosus tendon located above the medial meniscus. The patient is instructed to perform isometric contractions in knee flexion with the support of an assistant. If there is a ramp lesion, a low echoic space appears below the semimembranosus tendon and joint capsule. The medial meniscus left behind the contraction of the semimembranosus muscle can be observed. The white arrow indicates the ramp lesion. Left is the relaxed state, and right is the isometric contraction state. (MM, medial meniscus; SM, semimembranosus.)

**Fig 4:**
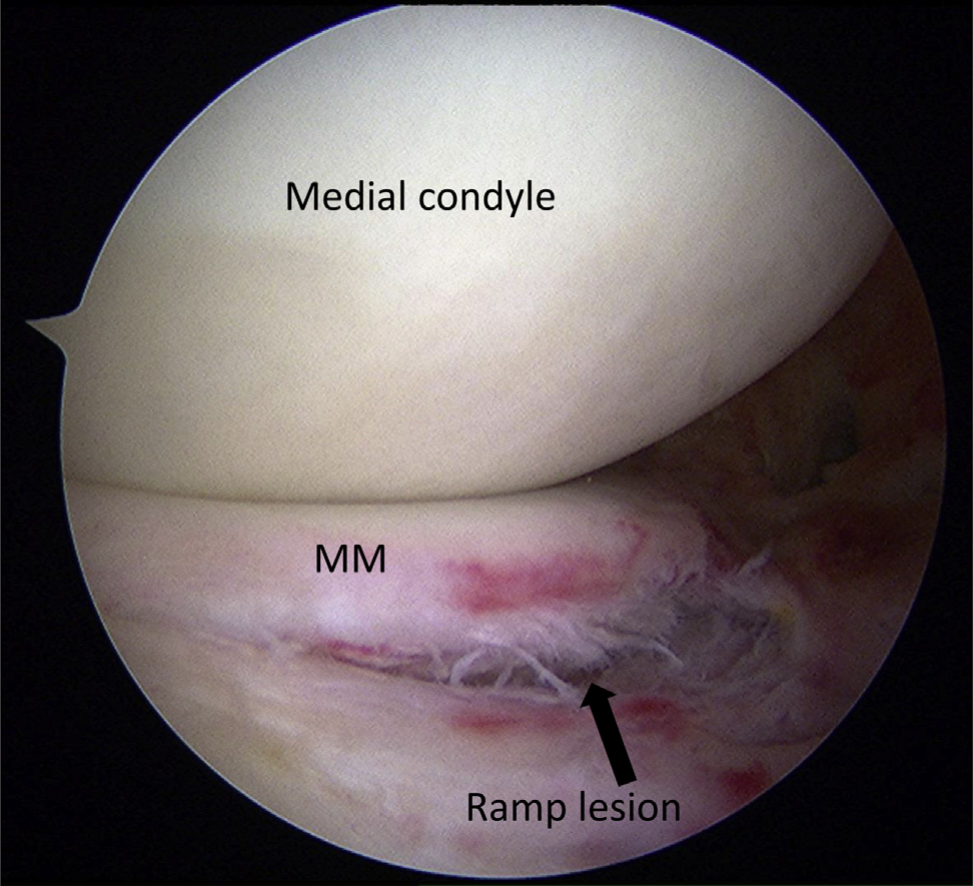
Arthroscopic findings from posteromedial portal of left knee joint. The black arrow indicates the ramp lesion. (MM, medial meniscus.)

The ultrasound technique is shown in Video 1. Diagnostic US was performed using the SONIMAGE HS-1 ultrasound system (Konica Minolta Healthcare, Tokyo, Japan) with a linear transducer (18-4 MHz). The patient is placed in the prone position with the knee flexed to approximately 70° and supported by an assistant (Fig 1). First, the semitendinosus tendon is palpated at the medial side of the popliteal fossa at 1 cm above knee joint line. A probe is placed just above the semitendinosus tendon to visualize the semitendinosus tendon and the semimembranosus muscle belly on the short axis view (Fig 2). The appearance resembles a “cherry on a pie.”^7^ The probe is then moved distally to visualize the semimembranosus tendon and the medial head of the gastrocnemius muscle belly (Fig 2). The probe is placed at approximately 90° just above the semimembranosus tendon to obtain a long-axis view. The semimembranosus muscle is a reliable landmark in this technique. The patient is instructed to perform isometric contractions in knee flexion with the support of an assistant. The examiner holds the probe firmly so that it does not shift. If there is a ramp lesion, a low echoic space appears below the semimembranosus tendon and joint capsule (Fig 3). The medial meniscus left behind the contraction of the semimembranosus muscle can be observed. We call this phenomenon the “meniscus left behind sign” (Fig 3). Fig 4 depicts the arthroscopic findings from a posteromedial portal view. The difference between the injured and healthy sides can be clearly confirmed, which is also one of the characteristics of US. The most important part of this technique is to instruct the patient to perform isometric contractions in knee flexion. Tips and tricks for identifying the ramp lesion are presented in Table 1, and the advantages and disadvantages of this technique are presented in Table 2. This report was reviewed and approved by the medical ethics review committee at the authors’ institution and was conducted in accordance with the Declaration of Helsinki. Informed consent was obtained from the patient, in both written and oral formats, regarding the report.

**Table 1:** Tips and Tricks for Identifying Ramp Lesions With Ultrasound Imaging

1	SAV	Put probe just above the semitendinosus tendon (cherry on a pie) knee flexed to approximately 70° in prone position.
2	SAV	Move probe distally to visualize SM tendon and medial head of gastrocnemius.
3	LAV	Rotate the probe approximately 90° parallel to the SM tendon.
4	LAV	Draw the medial meniscus in the deep layer of the SM tendon.
5	LAV	Instruct the patient to perform isometric contraction in knee flexion with the support of an assistant.
6	LAV	Check the “meniscus left behind sign.”
7		Compare with healthy side.

LAV, long-axis view; SAV, short-axis view; SM, semimembranosus.

**Table 2:** Advantages and Disadvantages

Advantages	Disadvantages
Evaluated by knee flexion position	Requires proficiency in the procedure
Evaluated dynamically	Not possible immediately after injury
Compared with healthy side	Need an assistant
Low cost	
Quick	

## Discussion

To visualize the ramp lesion preoperatively, it is important to keep the knee joint in a flexed position and for the patient to contract the semimembranosus muscle. US is the only imaging tool that can solve both of these problems. This Technical Note is the first report of the observation of ramp lesions by US.

Conventionally, MRI is the most commonly used imaging modality for the diagnosis of meniscal tears. A systematic review reported excellent specificity of MRI for diagnosing medial meniscus tears other than ramp lesions.^8^ In terms of patient knee position, ramp lesions are hardly visible in near-complete knee extension because those positions minimize the space between the MMPH and the capsule. In contrast, during knee flexion, the posterior capsule shifts posteriorly, and the separation in the meniscocapsular junction widens. We set the patient’s knee flexion angle to 70° instead of 90° to facilitate the examiner’s probe motion. Static observation in the flexed knee position did not reveal any ramp lesions. Furthermore, since external rotation of the lower leg enlarges the ramp lesion on arthroscopic evaluation, we also attempted internal and external rotation of the lower leg on US evaluation, but the ramp lesion could not be confirmed. US can evaluate dynamic motion, and when we instructed the patient to perform an isometric contraction in the knee flexion position, we were able to observe the ramp lesion clearly. The application of a load to the semimembranosus tendon resulted in posterior translation of the PHMM. If a ramp lesion is present, the medial meniscus will be left behind, unable to move posteriorly in conjunction with it. On US, a ramp lesion is a hypoechoic lesion, which is more easily identified on dynamic evaluation. The examiner should instruct the patient to perform several isometric contractions and fix the probe. It is important to confirm the continuity of the medial meniscus and the posterior joint capsule in the deeper layers of the semimembranosus tendon.

US is a nonirradiating, low-cost, real-time imaging modality that has good spatial resolution. US supplements MRI; it does not replace it. We use MRI to diagnose injuries such as ACL tears and conventional meniscus tears. US allows us to specifically assess for ramp lesions and test the knee dynamically. At our institution, an US is performed in every patient with an ACL tear. The clinical relevance of this report is that performing a dynamic US in every patient with an ACL tear can allow surgeons to detect hidden lesions preoperatively.

## Supplementary Data

Video 1 Ultrasound technique of a left meniscal ramp lesion. (LAV, long-axis view; mGC, medial head of gastrocnemius; MM, medial meniscus; SAV, short-axis view, SM, semimembranosus; ST, semitendinosus.)

ICMJE author disclosure forms
